# The complete mitochondrial genome of *Acanthogobius stigmothonus* (Perciformes, Gobiidae)

**DOI:** 10.1080/23802359.2020.1791004

**Published:** 2020-07-20

**Authors:** Jian Zheng, Weiye Gao, Zhicheng Sun, Tianxiang Gao, Na Song

**Affiliations:** a Key Laboratory of Mariculture, Ocean University of China, Ministry of Education, Qingdao, China; b Fishery College, Zhejiang Ocean University, Zhoushan, China

**Keywords:** Mitochondrial genome, *Acanthogobius stigmothonus*, phylogenetic

## Abstract

The complete mitochondrial genome of *Acanthogobius stigmothonus* is first sequenced in this study. This genome was 16,666 bp in size and contained 37 classic genes, including 13 protein-coding genes, 22 transfer RNA genes and two ribosomal RNA genes. The gene organization and nucleotide composition were the same with those found in most other Gobiidae fishes. Among 37 genes, 28 were encoded by the heavy strand, while nine were encoded by the light strand. The total nucleotide composition of this genome was 27.4% for adenine (A), 17.6% for guanine (G), 28.2% for cytosine (C), and 26.8% for thymine (T), with a high A + T content of 54.2%. This study will provide a better understanding of population genetic diversity of *A. stigmothonus* and offer useful information for future studies concerning Gobiidae mitogenome evolution.

*Acanthogobius stigmothonus* is a member of the family Gobiidae. It is distributed along the southern coastal area of China, which is endemic to China. *Acanthogobius stigmothonus* is an offshore warm water fish species which mainly inhabits in the shallow sea reef area (Wu et al. 2008). This species is similar to *Acanthogobius flavimanus* in morphological characters and is often misclassified (Shibukawa and Iwata 2013). *A. stigmothonus* is difficult to collect samples because of little catching amount. Few studies about *A. stigmothonus* were conducted until now, and no genetic studies was reported. Therefore, the complete mitochondrial genome of *A. stigmothonus* (GenBank: MT258987) is determined for the first time in this study.

One individual of *A. stigmothonus* was collected from the seacoast of Beihai, China, which was stored in 95% ethanol and deposited at the Fish Specimen Room of Fisheries Ecology Laboratory (specimen accession no. OUC-FEL202000327), Fisheries College, Ocean University of China, Qingdao city, Shandong Province, China. The total genomic DNA was extracted from *A. stigmothonus* muscle using traditional phenol-chloroform extraction method (Taggart et al. 1992). We then determined its complete mitochondrial genome sequence by polymerase chain reaction amplification and Sanger sequencing technology.

This mitochondrial genome was 16,666 bp in length, including 13 protein-coding genes, 22 tRNA genes, two ribosomal RNA genes, and a D-loop region. The structure and gene arrangement of this mitochondrial genome were identical to those obtained from other fish species such as *Acanthogobius hasta* (Kim et al. 2004) and *Larimichthys polyactis* (Cheng et al. 2012). Most of the genes of *A. stigmothonus* were encoded on the heavy strand (H-strand), except for NAD6 gene and eight tRNA genes encoded on the light strand (L-strand). The length of tRNAs ranged from 68–75 bp. Except for COI genes starting with guanine thiamine guanine (GTG), the remaining 12 protein-coding genes started with adenine thiamine guanine (ATG). The 12S-and the 16S ribosomal RNA were annotated with corresponding sizes of 952 and 1687 bp, respectively. The D-loop control region, with a full length of 982 bp, was located between transfer RNA (tRNA)-Pro and tRNA-Phe. The total nucleotide composition of this genome was 27.4% for A, 17.6% for G, 28.2% for C, and 26.8% for T, with a high A + T content of 54.2%. The total length of 13 protein-coding genes was 11,533 bp, and the nucleotide composition was 25.2% for A, 17% for G, 30.8% for C, and 27% for T.

The phylogenetic relationship of *A. stigmothonus* was analyzed using 13 concatenated protein-coding genes (Figure 1). A neighbor-joining (NJ) tree was constructed by software MEGA 5.0 (Pennsylvania, PA, USA). From the tree topologies, we can conclude that *A. stigmothonus* was genetically closest to species *A. hasta* (*Acanthogobius ommaturus*) (Song et al. 2010), and then to *Amblychaeturichthys hexanema* among 13 species within Gobioidei.

**Figure 1.**
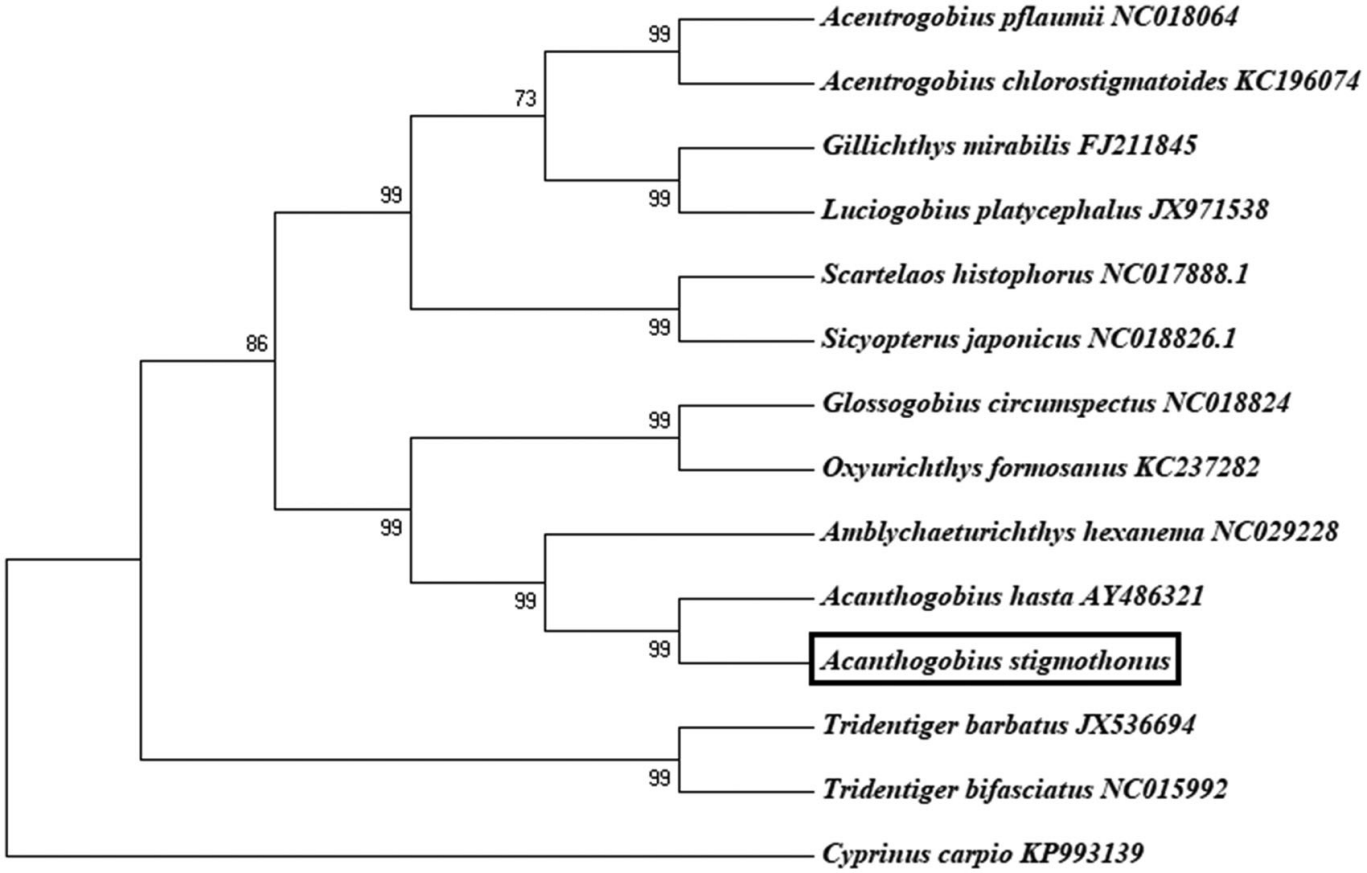
The phylogenetic analyses investigated using neighbor-joining (NJ) based on nucleotide sequences of 13 concatenated protein-coding genes. *Cyprinus carpio* (GenBank: KP993139) was used as an outgroup.

## Data Availability

The data that support the findings of this study are openly available in GenBank at https://www.ncbi.nlm.nih.gov/genbank/, accession number [MT258987].
